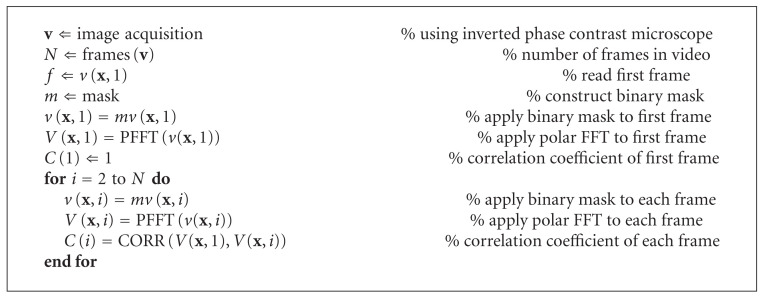# Image Processing Techniques for Assessing Contractility in Isolated Neonatal Cardiac Myocytes

**DOI:** 10.1155/2011/729732

**Published:** 2011-08-04

**Authors:** Carlos Bazan, David Torres Barba, Peter Blomgren, Paul Paolini

**Affiliations:** ^1^Computational Science Research Center, San Diego State University, 5500 Campanile Drive, San Diego, CA 92182-1245, USA; ^2^Computational Science Research Center, San Diego State University, 5500 Campanile Drive, San Diego, CA 92182-1233, USA; ^3^Department of Mathematics & Statistics, San Diego State University, 5500 Campanile Drive, San Diego, CA 92182-7720, USA; ^4^CardioMyocyte Dynamics Research Lab, Department of Biology, San Diego State University, 5500 Campanile Drive, San Diego, CA 92182-4614, USA

## Abstract

We describe a computational framework for the quantitative assessment of contractile responses of isolated neonatal cardiac myocytes. To the best of our knowledge, this is the first report on a practical and accessible method for the assessment of contractility in neonatal cardiocytes. The proposed methodology is comprised of digital video recording of the contracting cell, signal preparation, representation by polar Fourier descriptors, and contractility assessment. The different processing stages are variants of mathematically sound and computationally robust algorithms very well established in the scientific community. The described computational approach provides a comprehensive assessment of the neonatal cardiac myocyte contraction without the need of elaborate instrumentation. The versatility of the methodology allows it to be employed in determining myocyte contractility almost simultaneously with the acquisition of the Ca^2+^ transient and other correlates of cell contraction. The proposed methodology can be utilized to evaluate changes in contractile behavior resulting from drug intervention, disease models, transgeneity, or other common applications of neonatal cardiocytes.

## 1. Introduction

The analysis of cardiocyte mechanics has historically proven an excellent tool in providing relevant information on the excitation-contraction coupling of the heart [1]. It has provided useful insights toward the proper handling and treatment of many cardiovascular diseases. Furthermore, the study of cardiocyte contractility has helped unveil the fundamental processes underlying heart function in health and disease [2, 3]. The relevance of this study has created a need for analysis tools in this area of research.

The purpose of this paper is to propose a tool for the analysis of neonatal cardiocytes. Many inotropic factors modulate the contractile behavior of the heart which can be conveniently studied in isolated cardiocytes [1, 3–5]. Adult cardiac ventricular myocytes have been studied in cardiovascular research for almost thirty years, and the popularity of this approach is constantly reinforced by the numerous studies published every year [1]. However, during the last decade, the majority of researchers performing long-term (longer than 1 week) studies have favored the use of embryonic and neonatal cardiocytes [5]. This is due to their versatility and ability to withstand and survive harsher experimental conditions than adult cardiocytes.

Adult, neonatal, and embryonic cardiocytes are in different states of maturation and development. As the cardiac system of an organism matures, structural changes at the cellular level and in the myocardial anatomy occur to increase contractility and the development of force during contraction. It would be improper to perform studies on a myocyte at a given developmental level and make a direct correlation to another developmental stage, therefore ignoring the marked growth and structural differences that exist. Dramatic differences in action potential, physiology, gene expression, and molecular interactions in the neonatal and adult cardiocytes indicate that there is a need for different treatment of adult and newborn hearts [6]. It is therefore necessary to be able to perform equivalent studies on both adult and neonatal cardiocytes in both research and clinical applications.

Contractility—being the most representative of the functions of the cardiocyte—must be fully studied both quantitatively and qualitatively in all stages of development. It is important to identify and explore the differences in the adult and neonatal cardiocytes. It is thus desirable to have analysis methods that can be applied in a simple and practical manner to cardiocytes at all levels of development that will allow researchers to accomplish the aforementioned goals.

We have experienced the need for a practical and inexpensive—yet robust—method for assessing the contractility of neonatal and developing cardiocytes. Contractility assessment methods currently available to researchers leave room for improvement. This is particularly so in the case of the neonatal cardiocyte, in which a practical and generally applicable method is still missing. Because of this need, we propose a computational framework based on well-established image processing techniques for the assessment of contractility of isolated neonatal cardiac myocytes. The proposed methodology is easy to understand and implement, yet provides a robust account of the neonatal cardiocyte contraction process without the need for expensive and sophisticated equipment.

## 2. Background and Previous Work

**Figure 1:**
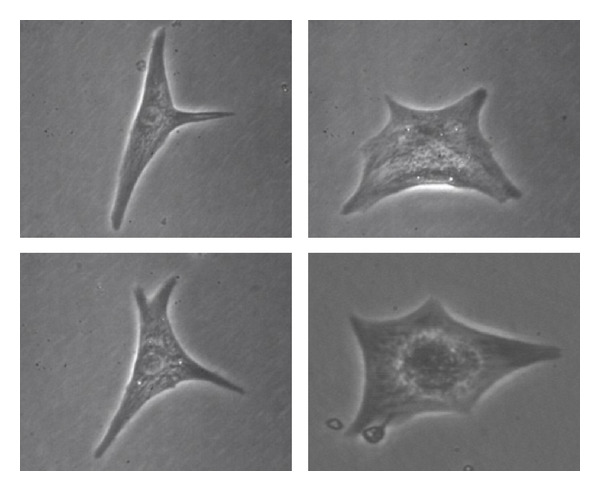
Four different neonatal cells after developing pseudopodia and spreading out on the substrate. They show almost unique shapes which greatly complicate the analysis of contractility.

Neonatal cardiocytes studied in the laboratory are generally harvested a few days after birth. In contrast to the adult cardiocyte, the neonatal cardiocyte is round in shape when first isolated from the heart [7]. Once the cells are plated, over time, they begin to form pseudopodia and spread out on the substrate, displaying a myriad of shapes [8] (see Figure 1). This diversity in morphology complicates the analysis of contractility. Development of myofibrils and striations occur as neonatal cardiocytes differentiate. These developments occur at different rates as they are highly dependent upon the composition of the culture media, isolation procedure, extracellular matrix (ECM), and species of the animal [5, 8–10].

Unlike adult cardiocytes which are highly organized and quite similar in morphology, the neonatal cardiocyte is in the process of developing their contractile machinery. The neonatal cardiocyte is generally unable to retract its cell boundary during contraction, and noticeable changes occur only within the cell perimeter. For these reasons, it is difficult to perform contractile measurements on this cell type in a manner similar to that of the adult cardiocyte, in which changes in cell boundary are quantified during contraction.

There are several methodologies for assessing the contractility of adult cardiocytes; however, very few methods have been proposed for the study of neonatal myocytes. The reported methods for quantifying or assessing neonatal cell contraction have required the use of elaborate equipment, such as a proximity detector or an atomic force microscope to measure the increase in cell elevation as a cardiocyte contracts [11]. Such contraction quantification and assessment methods are expensive due to the equipment needed. The application of the cell boundary video tracking method and the use of cellular force measurements using single-spaced polymeric microstructures to detect amplitude of contraction and beating rate in neonatal cardiocytes have also been reported in the literature [12–16]. These have often been found to be laborious, complicated, erratic, and expensive in laboratory studies. 

### 2.1. Fourier Transform and Image Analysis

We have recently explored the application of Fourier transform methods and image analysis techniques in the assessment of contractility in adult cardiocytes with satisfactory results [17]. Given our recent experience with this approach, the possibility of applying similar methods to the assessment of neonatal cardiocytes was obvious. Image analysis allows the application of investigation methods that are noninvasive and that can adapt to the different shapes and forms that a neonatal cardiocyte might take. Additionally, both the image analysis techniques and the Fourier transform are methods that can be applied without the need for expensive equipment or sophisticated mechanisms. This approach could potentially open the door to the possibility of applying similar methods for the assessment of contractility of cardiocytes in all stages of development, ranging from embryonic to adult hearts.

The correlation between myocyte contractile activities and the changes in intracellular calcium provide an insight into the mechanisms of calcium-dependent contraction. It is well known that Ca^2+^ is essential in cardiac electrical activity and that it is the direct activator of myofilaments in the cardiocyte, resulting in a contraction [18]. In adult ventricular myocytes, contractile activity is known to depend on the release of Ca^2+^ from the sarcoplasmic reticulum (SR) through ryanodine receptor channels (RyR), whereas in the fetal and neonatal myocytes, SR plays a much smaller role in Ca^2+^ regulation. This decreases the capability of these myocytes to load Ca^2+^ as compared to those isolated from mature hearts [19]. Fetal and neonatal contraction depends largely on trans-sarcolemmal Ca^2+^ influx rather than on Ca^2+^ released by the SR. This distinction translates into a slower upstroke and decay of Ca^2+^ in comparison with those of adult ventricular myocytes. This behavior is confirmed by a major difference between action potentials (AP) of adult and neonatal rat myocytes, with a significantly longer repolarization phase in neonatal cells as compared to those of adult myocytes [20]. We therefore anticipate neonatal cardiocyte contractility records to exhibit slower activation and relaxation periods as compared to those of the adult cardiac myocyte. Given the relationship that exists between Ca^2+^ and mechanical contractility, we also anticipate that the contractile behavior of neonatal cardiocytes would have strong correlation with the adult Ca^2+^ transient behavior. Given that a practical and accessible method for quantifying neonatal contractility has not been reported in the literature, we have decided to validate our results by comparing them to the contractile data of adult cardiocytes recently published in [17].

### 2.2. Neonatal Rat Ventricular Myocyte Isolation and Culture

Ventricular myocytes were isolated from 1–3-day-old Harlan Sprague-Dawley rats (*Rattus norvegicus albinus*), and cultured as described by Sprenkle et al. [21]. All animal procedures were in accordance with the San Diego State University Animal Subjects Committee (UASC) and NIH Animal Welfare Assurance A3728-01. After preplating myocytes to remove fibroblasts, pooled myocytes were centrifuged and resuspended in DMEM/F-12 (Gibco) containing 10% Fetal Bovine Serum (Irvine Scientific). Myocytes were then plated overnight on Fibronectin (Gibco)-coated 100 mm dishes to allow for recovery. Neonatal rat ventricular myocytes were then washed twice with 1 : 1 Medium consisting of DMEM/F-12, Kanamycin, Ampicillin, and Fungizone on the following day. Cells were then incubated overnight in a Minimal Medium consisting of 1 : 1 Medium, supplemented with 1 mg/mL BSA (Sigma). On the third day, cells were washed two more times with 1 : 1 Medium and replaced with an ITS Medium consisting of Minimal Medium, 1 X ITS (Insulin-Transferrin-Selenium, Gibco), 0.4 X MEM Nonessential Amino Acids mixture (Sigma), and 0.1 X MEM Vitamins medium (Gibco).

### 2.3. Image Acquisition

Images depicting contracting myocytes in this study were acquired using an inverted phase contrast microscope (Nikon number ELWD, Nikon Corporation, Tokyo, Japan). Myocytes with obvious sarcolemmal blebs or spontaneous contractions were not used. The cells were field stimulated with a suprathreshold (50%) voltage at a frequency of 0.3 Hz, for a 3 msec duration. The stimulation was performed using a pair of platinum wires placed on opposite sides of the chamber connected to an electrical stimulator (Hugo Sachs Elektronik-Harvard Apparatus, Type 223, Germany). The polarity of the stimulatory electrodes was reversed automatically every 10 stimuli to prevent electrode polarization. Myocyte motion was digitally recorded with a camera (PULNIX TM-1327, JAI PULNIX Inc., San Jose, CA, USA) mounted on the microscope, at a rate of 30 fps. Video files containing the contraction activities were stored for the analysis.

## 3. Assessment of Contractile Responses of Neonatal Cardiocytes

Two popular shape descriptor methodologies used in image processing are contour-based and region-based descriptors. We have applied contour-based descriptors for the assessment of contractile responses of isolated adult cardiac myocytes [17]. Such descriptors assume the knowledge of shape boundary information and are usually suitable for characterizing contour shapes without sophisticated boundaries such as those of the adult cardiocyte. Region-based shape descriptors—specifically the intensity-based descriptors—can be applied to more general cases where the contractile responses do not necessarily provoke changes in the overall geometry of the cell, but rather the rearrangement of its interior structures. This is the case of the neonatal cardiac myocyte. In this section we describe a method for assessing the contractions of neonatal cardiocytes by analyzing Fourier descriptors obtained through (discrete) Polar Fourier transform (PFT).

Fourier descriptors have been extensively proposed for the purpose of shape recognition, retrieval, classification, and analysis [22–30]. One of the main advantages of analyzing images in the spectral domain—rather than analyzing them in the spatial domain—is that it is easier to overcome the noise problem common to digital images. Furthermore, the spectral features of an image are usually more concise than the features extracted from the spatial domain [31]. Although, the direct application of the (discrete) Fourier transform (FT) to an image can provide useful information about its content, it has the disadvantage of lacking rotation invariance. The PFT generates rotation-invariant data particularly well suited for effective extraction of orientation features.

The following description of the PFT is based on the works by Averbuch et al. [32–35]. They proposed a Polar Fast Fourier transform (PFFT) method by using a special type of Unequally Spaced Fast Fourier transform (USFFT), where a different starting grid is employed, instead of the regular Cartesian grid. Their methodology cleverly decomposes the problem into two stages: first, a pseudo-Polar sampling set is used to apply a pseudo-Polar FFT, and second, a conversion from pseudo-Polar to Polar FT is performed. This approach provides improved performance as compared to the alternative Cartesian-based USFFT-based counterparts. The main reason for this improvement is in the ability of the pseudo-Polar grid to provide a spatial-varying sampling of the frequency domain, which is closer in density to the final Polar grid. Furthermore, the pseudo-Polar grid gives a denser sampling near the origin, allowing for a more accurate interpolation.

**Figure 2:**
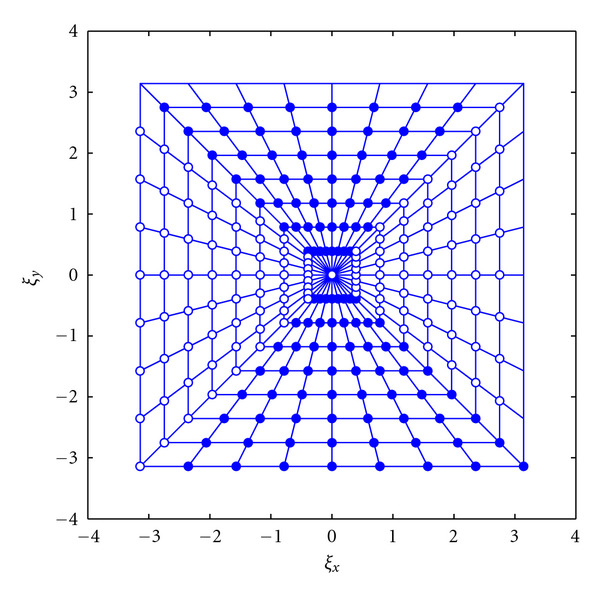
Basically vertical subnet (filled circles) and basically horizontal subnet (hollow circles) comprising the pseudo-Polar grid formed by the intersection of 8 concentric squares and 16 slope-equispaced rays. (This figure first appeared in [33]).

As mentioned above, the pseudo-Polar Fourier transform based on the definition of a Polar-like 2D grid provides a fast Fourier computation (see Figure 2). Averbuch et al. [33] (and more recently in [34, 35]) built their methodology upon their work on Radon Transform for data in a Cartesian grid [32], based on the summation along lines of absolute slope less than one, with values at non-Cartesian locations which are defined using trigonometric interpolation on a zero-padded grid. Their implementation of the polar FFT starts by defining the pseudo-Polar grid points in the frequency domain. There are two types of points on the grid: the basically vertical (BV) subnet and the basically horizontal (BH) subnet. These are expressed as follows:

(1) $$\begin{array}{c} \text{BV}=\left\{\begin{array}{cc} \xi_{y}=\frac{\pi l}{N} & \text{for}-N⩽l<N,\\ \xi_{x}=\frac{2\pi ml}{N^{2}} & \;\;\;\;\;\;\text{for}-\frac{N}{2}⩽m<\frac{N}{2} \end{array}\right\}, \end{array}$$

(2) $$\begin{array}{c} \begin{array}{c} \text{BH}=\left\{\begin{array}{cc} \xi_{x}=\frac{\pi l}{N} & \text{for}-N\leq l<N,\\ \xi_{y}=\frac{2\pi ml}{N^{2}} & \;\;\;\;\;\;\text{for}-\frac{N}{2}<m⩽\frac{N}{2} \end{array}\right\} \end{array} \end{array}.$$

The pseudo-Polar grid is shown in Figure 2 where the BV points are marked with the filled circles, while the BH points are marked with hollow circles. Averbuch et al. [33] have shown that given the pseudo-Polar grid points BV and BH, in order to compute the Fourier transform values, a simple 1D-FFT can be satisfactorily employed.

Based on the aforementioned pseudo-Polar coordinates system, the Polar coordinate system is defined by manipulating the layout through appropriate interpolations. The two operations involved in this transformation are to rotate the rays to obtain an angularly-uniform ray sampling and circle the squares to obtain concentric circles as required in the Polar coordinate system. To rotate the rays, the term 2*m*/*N* is replaced with tan(*πm*/2*N*) in *ξ*_*x*_ and *ξ*_*y*_ in (1) and (2), respectively. That will lead to the new grid points:

(3) $$\begin{array}{cc} \text{B}{\text{V}}_{\text{new}} & =\left\{\begin{array}{cc} \xi_{y}=\frac{\pi l}{N} & \text{for}-N⩽l<N,\\ \xi_{x}=\frac{\pi l}{N}\tan\left(\frac{\pi m}{2N}\right) & \;\;\;\;\;\text{for}-\frac{N}{2}⩽m<\frac{N}{2} \end{array}\right\},\\ \text{B}{\text{H}}_{\text{new}} & =\left\{\begin{array}{cc} \xi_{x}=\frac{\pi l}{N} & \text{for}-N⩽l<N,\\ \xi_{y}=\frac{\pi l}{N}\tan\left(\frac{\pi m}{2N}\right) & \;\;\;\;\;\text{for}-\frac{N}{2}<m⩽\frac{N}{2} \end{array}\right\}. \end{array}$$

**Figure 3:**
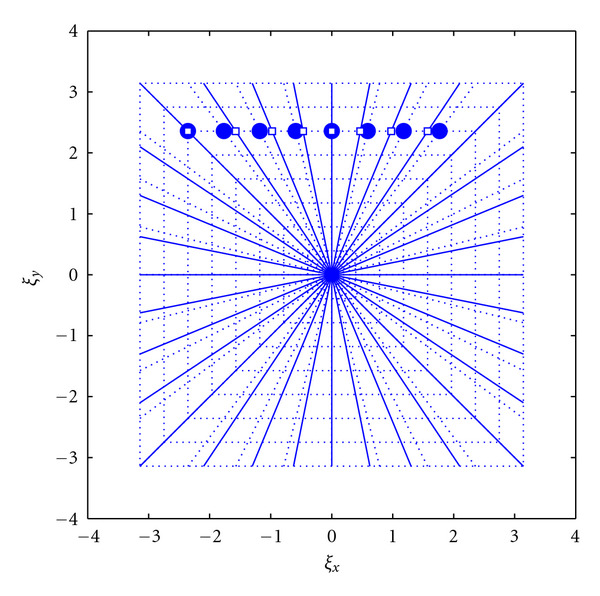
Rotate the rays interpolation stage. Circles denote the known pseudo-Polar grid points while the squares are the destination equiangular spaced rays. (This figure first appeared in [33]).

The new points are still organized in concentric squares, but the rays are now equispaced based on angle as opposed to slope (see Figure 3).

To circle the squares, both *ξ*_*x*_ and *ξ*_*y*_ are divided by a constant along each ray, based on its angle, and thus a function of the parameter *m*:

(4) $$R\left(m\right)={\left(1+\text{ta}{\text{n}}^{2}\left(\frac{\pi m}{2N}\right)\right)}^{1/2}.$$

The resulting grid is expressed as follows:

(5) $$\begin{array}{c} \xi_{y}=\frac{\pi l}{NR\left(m\right)},\;\text{for}-N⩽l<N,\\ \xi_{x}=\frac{\pi l}{NR\left(m\right)}\tan\left(\frac{\pi m}{2N}\right)\;\text{for}-\frac{N}{2}⩽m<\frac{N}{2}. \end{array}$$

**Figure 4:**
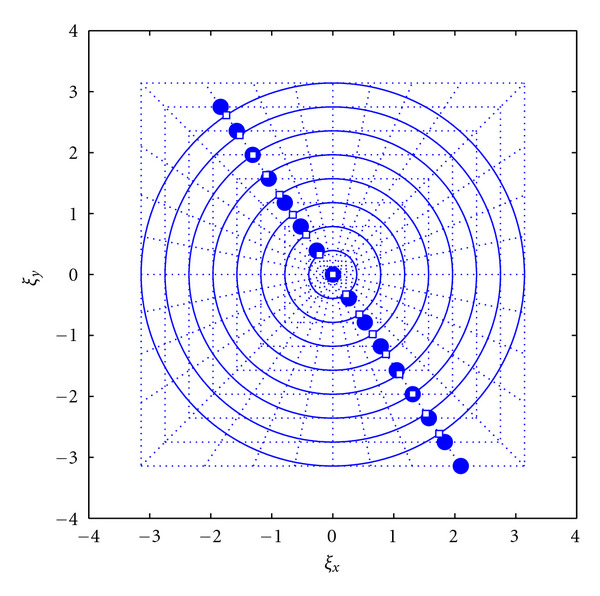
Circle the squares interpolation stage. Circles denote the known pseudo-Polar grid points while the squares are the destination equiangular spaced rays. (This figure first appeared in [33]).

**Figure 5:**
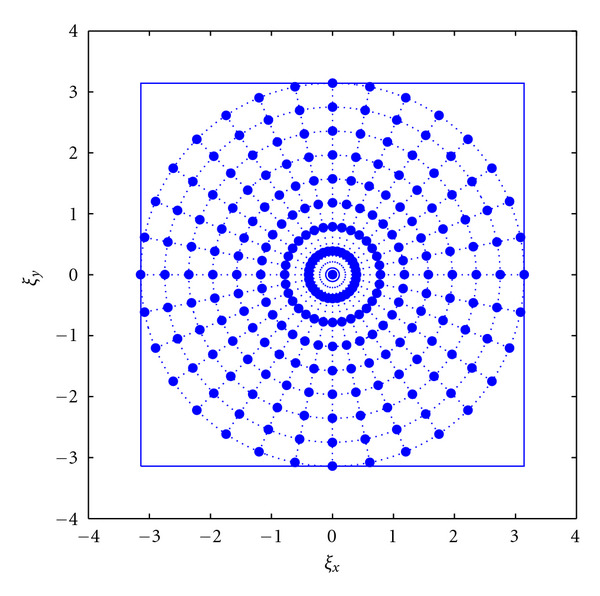
Final Polar grid points after both interpolation processes have been applied. (This figure first appeared in [33]).

The new points are located along the same line and are equispaced with a different spacing (see Figure 4). The final grid is shown in Figure 5.

**Figure 6:**
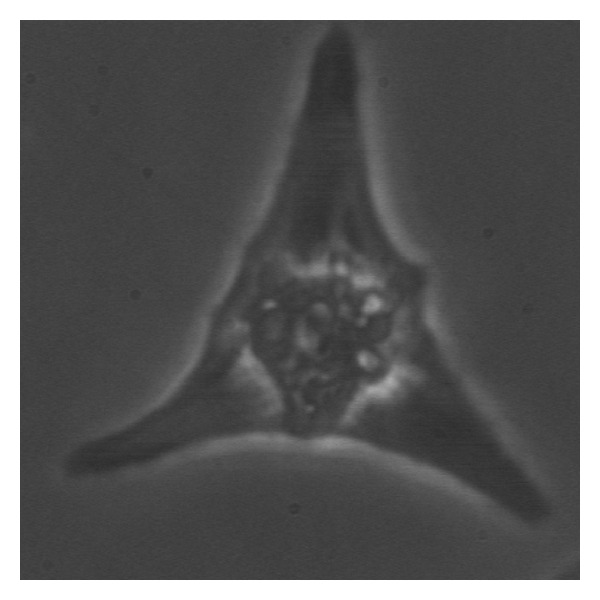
Image of neonatal cardiac myocyte observed by using phase contrast microscope and captured by employing a high-definition digital camera.

**Figure 7:**
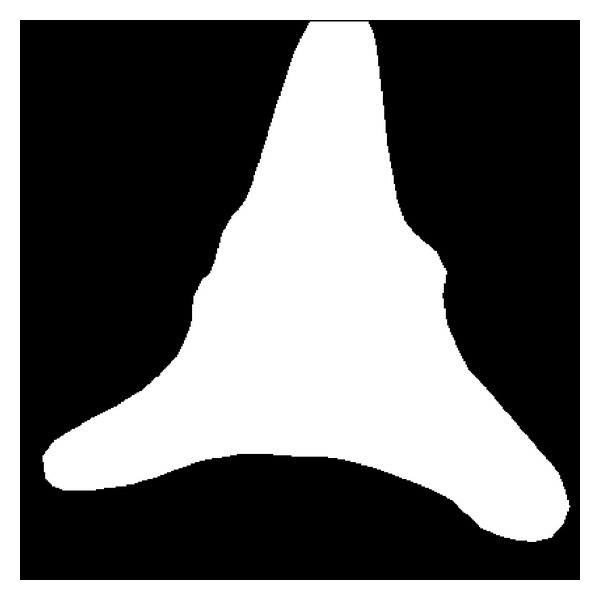
Mask used to constrain the analysis to the cardiac myocyte contractile responses. The mask is used to restrict the analysis to regions of interest.

In order to prevent wrap around and thus to get geometrically faithful lines, the pseudo-Polar grid must be oversampled both radially and angularly. In their implementation, Averbuch et al. [33] apply the methodology to general images of size *N* × *N* and obtained a resulting Polar FT array of 2*N* + 1 × 2*N* + 1 (the authors thank reviewer for pointing this out). They argued that, if higher oversampling is desired in the destination grid, an easy way of accomplishing this is to modify the initial value *N* by zero-padding the input array. For measuring the contractile responses in neonatal cardiocytes, we zero-pad each frame with a mask that isolates the cell from the background, while at the same time, it turns each frame into an *N* × *N* image suitable for applying the above methodology.  Figure 6 shows a frame of a neonatal cardiocyte that has been cropped to size *N* × *N*.  Figure 7 shows the *N* × *N* mask that is used to isolate the region of interest for the analysis.

**Figure 8:**
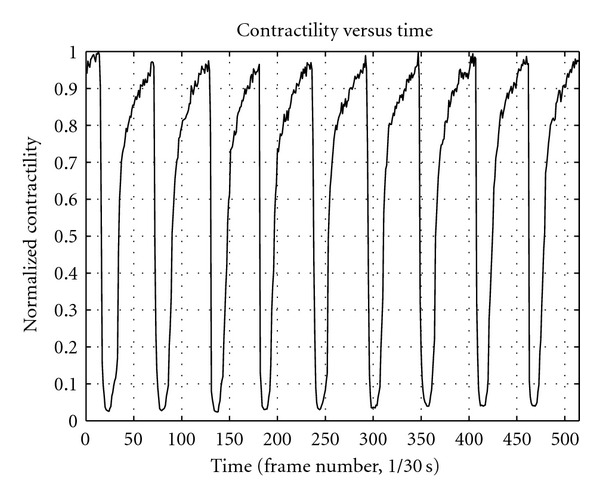
Neonatal cardiac myocyte contractile responses obtained by employing the neonatal contractility assessment method proposed in this paper.

Cross-correlation of the PFT from each image (frame) is used to calculate a statistical estimation of the similarities between image components around an interest point of the cardiocyte. This provides an account of the contractile responses of the cardiocyte to the electrical stimulus. Cross-correlation is a very simple method which, if used in the proposed manner, provides a robust measurement of the changes manifesting as contractions occur.  Figure 8 depicts the data of nine consecutive contractions occurring in a neonatal cardiocyte. It is important to note the regularity of the contraction signal as well as the great signal-to-noise ratio that is obtained using this method.

In Algorithm 1, we present a simplified algorithm for the assessment of contractile responses of neonatal cardiocytes (for details on the implementation of the polar FFT the reader is referred to [33].

## 4. Experimental Results

Results obtained by applying the proposed image processing techniques for assessing contractility in isolated neonatal cardiac myocytes proved to be satisfactory. We observe a contractile response of the neonatal cardiocyte that is in accordance with structural and functional differences known to exist between adult and neonatal cardiac myocytes.

Given the relationship that exists between Ca^2+^ transient levels and contractility, we expected the neonatal contractile and Ca^2+^ transient behavior to have a correlation similar to the one that exists between the adult Ca^2+^ transient and its contractile behavior. Adult cardiac myocytes are known to exhibit Ca^2+^ transients that have a faster activation and recovery phase than that of the neonatal cardiocyte. This could be attributed to the fact that the contractile activity of adult ventricular myocytes is known to depend on the release of Ca^2+^ from the SR, whereas in the fetal and neonatal myocytes the SR plays a smaller role in Ca^2+^ regulation, decreasing the capability of these myocytes to load Ca^2+^ [19]. Excitation-contraction mechanisms of the fetal and neonatal myocytes are therefore more dependent upon extracellular Ca^2+^ to activate the contractile machinery, which is a slow-acting mechanism compared to that of the SR. The contractile proteins, cellular structures, and myofibrils of the adult cell are highly organized and developed. These factors result in a well-coordinated contractile process that translates into fast contraction and relaxation phases during contractility. In contrary, the neonatal cardiocyte has contractile proteins, cellular structures, and myofibrils in the developmental process [8], with an excitation-contraction coupling mechanism that is highly dependent on extracellular calcium. This translates into a slow and disorganized (semiasynchronous) activation-contraction mechanism.

**Figure 9:**
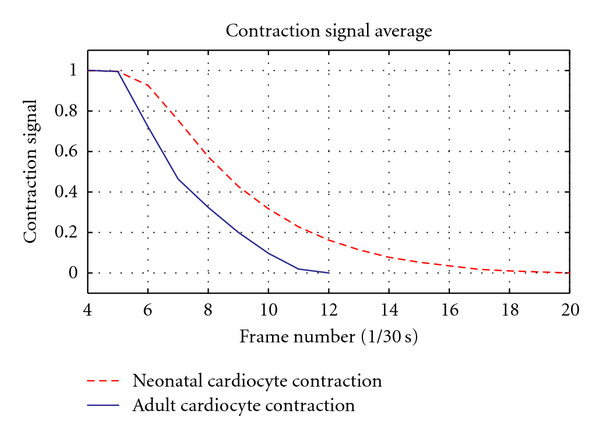
Solid line: average adult cardiocyte relaxation phase measured using image processing-based techniques recently proposed in [17]. Dashed line: average neonatal cardiocyte relaxation phase measured using the proposed methodology. Results are in accordance with the expected outcomes given the marked differences in the development of both cell types and their difference in Ca^2+^ handling.

**Figure 10:**
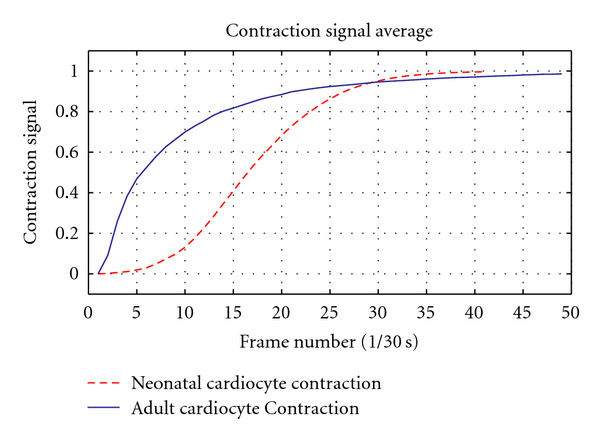
Solid line: average adult cardiocyte contraction measured using image processing-based techniques recently proposed in [17]. Dashed line: average neonatal cardiocyte contraction measured using the proposed methodology. Results are in accordance with the expected outcomes given the marked differences in the development of both cell types and their difference in Ca^2+^ handling.

**Figure 11:**
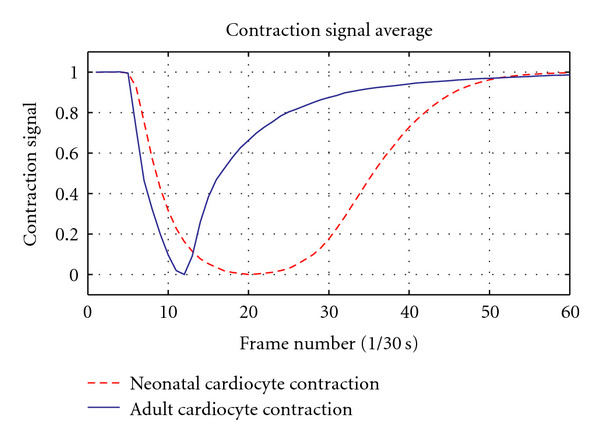
Solid line: Average adult cardiocyte contraction phase measured using image processing based techniques recently proposed in [17]. Dashed line: Average neonatal cardiocyte contraction phase measured using the proposed methodology. Results are in accordance with the expected outcomes given the marked differences in the development of both cell types and their difference in Ca^2+^ handling.

Based on these facts we anticipated the neonatal cardiocyte would exhibit signals with slower contraction and relaxation phases and a total contraction signal considerably weaker than that of the adult cardiocyte. Our results corroborate the anticipated outcome by exhibiting a clear correlation between the known Ca^2+^ transient of a neonatal cardiocyte and the results of our contractility assessment. The activation (contraction) phase is noticeably slower than that of the adult cardiocyte (see Figure 9), yet the most significant difference occurs in the relaxation phase, where there is a dramatic difference in the rate at which the neonatal cardiocyte mechanisms return to their basal conditions (see Figure 10). We also register a considerably long plateau at the peak of contraction, which can be attributed to the disorganization in the developing myofibrils—contracting in an asynchronous fashion—with their peaks of contraction occurring at different times (see Figure 11).

The resulting contractile signals from our preliminary results are in accordance with our expectations, exhibiting slower activation and relaxation rates as compared to those of the adult cardiac myocyte (see Figures 9 and 10). The total contraction exhibited by the neonatal cardiocyte is considerably less than the contractility exhibited by the adult cardiocyte (see Figure 11).

## 5. Discussion

We described a computational framework for the assessment of contractile responses of isolated neonatal cardiac myocytes. The assessment stages are based on mathematically sound and computationally robust algorithms very well established in the scientific community. Image analysis provides an opportunity to analyze cellular dynamics without disrupting the cellular environment in vitro. Our intent was to establish an accessible approach to assess neonatal contractility by making use of the methods available in the field of image analysis. We feel that the development of this methodology will provide scientists working in the field of cellular cardiology with a practical yet robust option to assess the contractility of neonatal cardiocytes. To our knowledge this is the first practical and easy to implement methodology that has been reported in the literature. The method captures the full extent of the contractile signal, which represents an important contribution to the analysis of cardiocytes and potential to cardiovascular research.

Our future work entails the use of this methodology in a real-time application, which can be used to analyze the contractility of neonatal cardiocytes under different conditions. We have recently published a similar image analysis-based application for the assessment of adult cardiac myocytes. Our intent is the creation of an image analysis-based methodology that can be used for assessing the contractility in cardiocytes in all stages of development and that can provide a reliable alternative to the commercially available contractility analysis tools. The availability of a similar protocol for measuring both neonatal and adult cardiocyte contractions would allow a consistent approach to characterize inotropic effects of drugs on cell types in all stages of development.

## Figures and Tables

**Algorithm 1:**